# Deterioration of Parkinson's disease during hospitalization: survey of 684 patients

**DOI:** 10.1186/1471-2377-12-13

**Published:** 2012-03-08

**Authors:** Oliver HH Gerlach, Martijn PG Broen, Peter HMF van Domburg, Ad J Vermeij, Wim EJ Weber

**Affiliations:** 1 Section of Movement Disorders, Department of Neurology, Maastricht University Medical Centre, Maastricht, The Netherlands; 2 Section of Movement Disorders, Department of Neurology, Orbis Medical Centre, Sittard-Geleen, The Netherlands; 3 Section of Movement Disorders, Department of Neurology, Catharina Hospital Eindhoven, Eindhoven, The Netherlands

## Abstract

**Background:**

A substantial fraction of Parkinson's disease patients deteriorate during hospitalisation, but the precise proportion and the reasons why have not been studied systematically and the focus has been on surgical wards and on Accident & Emergency departments. We assessed the prevalence and risk factors of deterioration of Parkinson's disease symptoms during hospitalization, including all wards.

**Methods:**

We invited Parkinson's disease patients from three neurology departments in The Netherlands to answer a standardised questionnaire on general, disease and hospital related issues. Patients who had been hospitalized in the previous year were included and analysed. Possible risk factors for Parkinson's disease deterioration were identified. Proportions were analysed using the Chi-Square test and a logistic regression analysis was performed.

**Results:**

Eighteen percent of 684 Parkinson's disease patients had been hospitalized at least once in the last year. Twenty-one percent experienced deterioration of motor symptoms, 33% did have one or more complications and 26% had received incorrect anti-Parkinson's medication. There were no statistically significant differences for these variables between admissions on neurologic or non-neurologic wards and between having surgery or not. Incorrect medication during hospitalization was significantly associated with higher risk (OR 5.8, CI 2.5-13.7) of deterioration, as were having infections (OR 6.7 CI 1.8-24.7). A higher levodopa equivalent dose per day was a significant risk factor for deterioration. When adjusting for different variables, wrong medication distribution was the most important risk factor for deterioration.

**Conclusions:**

Incorrect medication and infections are the important risk factors for deterioration of Parkinson's disease patients both for admissions with and without surgery and both for admissions on neurologic and non-neurologic wards. Measures should be taken to improve care and incorporated in guidelines.

## Background

Parkinson's disease (PD) patients are admitted to hospitals more frequently and longer than the general population [1,2]. Up to a quarter of the total PD patients are hospitalized each year [1]. There is general consensus that a substantial fraction of these hospitalized PD patients do deteriorate, but the precise proportion and the reasons why have not been studied systematically and the focus has been on surgical wards and on Accident & Emergency departments [3-6]. We found that, although many PD patients seem to deteriorate during hospitalization and there is concern about the quality of care provided to these patients [7], most hospitals do not have proper guidelines yet to prevent worsening of PD symptoms and complications during hospitalization [8]. Before such guidelines can be formulated, a better understanding of the problems encountered during hospitalization of this group of patients is warranted. Our aim in this study was to assess the prevalence and risk factors of deterioration in hospitalized PD patients including all wards.

## Methods

PD patients from 3 neurology departments in the southern part of The Netherlands were invited to participate in the survey, i.e. the Maastricht University Medical Centre in Maastricht, Orbis Medical Centre in Sittard-Geleen, and Catharina Hospital Eindhoven in Eindhoven. Only PD patients, of whom the diagnosis had been confirmed by a neurologist according to the UK Brain Bank criteria were selected. All patients with other or unclear parkinsonisms were excluded. The selected patients were sent a questionnaire by mail. This questionnaire consisted of questions concerning general, personal and disease related issues (see additional file 1 and 2). Patients were asked whether or not having cognitive problems. The obtain more accurate data, we asked patients to fill in the questionnaire with the help of a caregiver. Patients who confirmed that they had been admitted to a hospital in the previous year, were asked to answer more detailed questions about this hospital stay (e.g. exact timing or lack of drug administration, complications, and PD deterioration). After 4 weeks we sent a reminder to patients who had not yet returned the questionnaire. We validated the data by comparing the questionnaire-replies with corresponding hospital records. Only patients with a hospital submission in the previous year were included and analysed. Admissions for PD related brain surgery were excluded. Subsequently, we tried to identify possible risk factors for PD deterioration.

PD deterioration we defined as decline in motor function. Receiving incorrect PD medication during the hospital stay was defined as administration of PD drugs during the hospital stay not as home schedule with attention to interruption, wrong timing, and different PD medication. Levodopa Equivalent Dose (LED) was used to calculate the amount of anti-parkinsonian drugs [9].

The ethics committees of the 3 collaborating hospitals approved our study: Medical Ethics Committee academic hospital Maastricht/Maastricht university (reference number 08-5-082), Local Advisory Group Scientific Research Orbis Medical Centre (reference number 10.029), and Medical Ethics Committee Catharina Hospital Eindhoven (reference number M11-015). Research was carried out in compliance with the Helsinki Declaration.

### Statistical methods

We compared proportions using the Chi-Square test for independence and subsequently performed a logistic regression analysis. A *P*-value of less than 0.05 is considered statistically significant. Admissions were not included if there were data missing required for that specific analysis. All statistical analysis are performed with PASW-version 18.0 (SPSS, Chicago).

## Results

### Response rate

We invited 884 patients to participate, and data from 684 patients (response rate 77%) were available for this study (Table 1). In total 123 patients were admitted to hospital in the previous year, accounting for 159 admissions, and these were used for analysis. 60% of the PD patients filled in the questionnaire together with a caregiver.

**Table 1:** Patient and hospitalization characteristics

		MUMC		OMC		CHE		Total		Homogeneity^$^
**Total questionnaires (N)**		447		230		207		884		
**Response rate (%)**		72		84		81		77		
**Admitted patients (N)**		53		34		36		123		
**Total hospitalizations (N)**		61		47		51		159		
**Age (yr)**		71 [SD = 10.4]		75 [SD = 7.6]		71 [SD = 8.5]		72 [SD = 9.2]		0.87
**Disease duration (yr)**		9.6 [SD = 7.0]		9.1 [SD = 7.2]		10.7 [SD = 6.9]		9.8 [SD = 7.0]		0.49
**LED-value (mg/day)**		554		761		855		711		0.004
		N*	%*	N*	%*	N*	%*	N*	%*	
**Gender**										
	Women	28	*46*	15	*32*	14	*28*	57	*36*	0.04
	Men	33	*54*	32	*68*	37	*72*	102	*64*	
**Hoehn&Yahr**										
	stage < III	24	*39*	20	*43*	13	*25*	57	*36*	0.12
	stage III, IV	33	*54*	25	*53*	33	*65*	91	*57*	0.32
	stage V	3	*5*	2	*4*	5	*10*	10	*6*	0.33
	Don't know/missing	1	*2*	0	*0*	0	*0*	1	*1*	
**On-off fluctuations**										
	Yes	27	44	14	30	21	41	62	39	0.61
	No	33	54	33	70	30	59	96	60	
	Don't know/missing	1	2	0	0	0	0	1	1	
**Cognitive problems**										
	Yes	20	33	24	51	29	57	72	46	0.009
	No	41	67	23	49	22	43	86	54	
	Don't know/missing	0	0	0	0	0	0	0	0	
**Deterioration during admission**										
	Yes	13	*21*	10	*21*	11	*22*	34	*21*	0.84
	No	45	*74*	30	*64*	35	*69*	110	*69*	
	Don't know/missing	3	*5*	7	*15*	5	*10*	15	*9*	
**Complications during admission**										
	None	40	*66*	30	*64*	35	*69*	105	*66*	0.76
	One or more complications	21	*34*	15	*32*	16	*31*	52	*33*	0.30
	Confusion	13	*21*	11	*23*	11	*22*	35	*22*	0.91
	Urinary tract infection	4	*7*	6	*13*	2	*4*	12	*8*	0.70
	Emotional disturbance	6	*10*	0	*0*	0	*0*	6	*4*	0.004
	Pneumonia	0	*0*	2	*4*	2	*4*	4	*3*	0.17
	Memory complaints	2	*3*	0	*0*	3	*6*	5	*3*	0.50
	Falls	0	*0*	2	*4*	0	*0*	2	*1*	0.87
	Other	2	*3*	0	*0*	3	*6*	5	*3*	0.50
	Don't know/missing	0	*0*	2	*4*	0	*0*	2	*1*	
**Medication distribution**										
	Good	40	*66*	29	*62*	39	*76*	108	*68*	0.15
	Bad	18	*30*	16	*34*	8	*16*	42	*26*	
	Don't know/missing	3	*5*	2	*4*	4	*8*	9	*6*	
**Surgery**										
	Yes	39	*64*	21	*45*	31	*61*	91	*57*	0.63
	No	22	*36*	26	*55*	20	*39*	68	*43*	
	Don't know/missing	0	*0*	0	*0*	0	*0*	0	*0*	
**Ward**										
	Neurologic	11	*18*	6	*13*	11	*22*	28	*18*	0.68
	Non-neurologic	48	*79*	41	*87*	39	*76*	128	*81*	
	Don't know/missing	2	*3*	0	*0*	1	*2*	3	*2*	
**Involvement of paramedics**										
	Yes	23	*38*	29	*62*	8	*16*	60	*38*	0.025
	No	34	*56*	14	*30*	41	*80*	89	*56*	
	Don't know/missing	4	*7*	4	*9*	2	*4*	10	*6*	
**Consultation of PD nurse specialist^#^**										
	Yes	14	*23*	20	*43*	5	*10*	39	*25*	0.14
	No	45	*74*	24	*51*	44	*86*	113	*71*	
	Don't know/missing	2	*3*	3	*6*	2	*4*	7	*4*	

* N; number and percentage of total admissions

^# ^Non-neurological ward

$ For homogeneity between different centres the Pearson Correlation and Spearman's rho tests were used. P-values are shown. *P-*value < 0.05 is considered significant.

Abbreviations: MUMC, Maastricht University Medical Centre; OMC, Orbis Medical Centre; CHE, Catharina Hospital Eindhoven; N, number; SD, standard deviation; LED-value, Levodopa Equivalent Dose; PD, Parkinson's disease

### Hospitalization

Eighteen percent of the PD patients were hospitalized at least once in the last year with an average of 1.3 (ranging between one and four) admissions per patient per year. Patients were admitted most frequently on a non-neurological ward, being surgery (24%), internal medicine (22%), orthopaedics (15%), urology (13%), cardiology (11%) and others. Admission reasons for these wards were traumatic injury whether or not following surgery (20%), urinary tract problems (15%), gastrointestinal problems (15%), cardiac problems (12%), other surgical procedures (11%), elective joint replacement due to arthrosis (7%), pneumonia (6%), and others. Eighteen percent of the patients were admitted to a neurological ward. Of those, 71% had PD related problems (45% PD medication problems, 20% deterioration of PD, 10% PD related screening, 5% hallucinations/confusion, 5% swallowing problems, 15% unknown). Other reasons for admission to a neurological ward were mainly strokes.

More than a fifth of all patients experienced deterioration of motor PD symptoms during their hospital stay. Forty-four percent of them showed no complete recovery after discharge. Most patients stated to have an overall worsening of motor function (38%) or motor skills (32%). The other ones had a worsening of rigidity (12%), tremor (9%), balance problems (3%), or bradykinesia (3%).

For the group of patients that were admitted because of PD deterioration, one patient further deteriorated during this admission. This patient didn't receive correct PD medication.

A third of the patients did have one or more complications during the admission, mainly confusion followed by infections. Complications didn't differ between non-neurologic and neurologic wards (P = 0.83). There was not more confusion (P = 0.80) or other statistically significant differences in complication rates among patients whether or not having surgery. Of the patients having an infection as a complication during admission, non of them had an infection as admission reason.

More than a quarter of the patients reported receiving incorrect PD medication during the hospital stay, i.e. wrong timing (79%), different PD medication (29%) or interruption of PD medication (5%). No difference in medication distribution problems between neurologic and non-neurologic wards (P = 0.49) or whether or not patients having surgery (P = 0.07) was found. In 3% there was self-administration of PD drugs.

### Deterioration and relating factors

With respect to the general and PD related characteristics only for patients with a LED-value of more than 700 mg/day there is a significantly increased risk for deterioration of PD symptoms (Table 2).

**Table 2:** Effect of patient, Parkinson's disease characteristics, and factors during hospitalization on deterioration of Parkinson's disease

	Deterioration (N = 34)		
**Possible risk factors**	***N***	P-value*	***OR [95%-CI]***
**Gender**			
Male	25	0.39	
**Age**			
≥ 70 years	20	0.42	
≥ 80 years	5	0.47	
≥ 85 years	5	0.13	
**Disease duration**			
≥ 8 years	17	0.82	
≥ 10 years	9	0.18	
≥ 12 years	8	0.36	
**Hoehn&Yahr**			
stage ≥ II	27	0.50	
stage ≥ III	27	0.07	
**On-off fluctuations**	16	0.38	
**Cognitive problems**	19	0.18	
**LED-value**			
> 500 mg/day	15	0.60	
> 600 mg/day	15	0.07	
> 700 mg/day	15	0.003	4.4 [1.7-11.5]
**Complications ≥ 1**	16	0.04	2.5 [1.1-5.6]
Confusion	10	0.23	
Infections	7	0.00	6.7 [1.8-24.7]
**wrong medication distribution**	18	0.00	5.8 [2.5-13.7]
**surgery**	17	0.26	
**Non-neurologic ward**	27	0.60	
**No involvement of paramedics**	16	0.15	
**No-consultation of PD nurse specialist#**	17	0.04	0.3 [0.1-0.7]

*P-value < 0.05 is considered significant

^# ^Non-neurological ward

Abbreviations: N, number; OR, Odds-ratio; 95%-CI, 95%-confidence interval; LED, Levodopa equivalent dose; PD, Parkinson's Disease

As to hospital related risk factors incorrect medication administration during hospitalization was significantly associated with deterioration during admission. This was also the case when one or more complications occurred. Analysing the individual complications, only infections showed to be an significantly increased risk factor. No other variables were significant.

In 14% of the admissions, PD patients had both cognitive problems and didn't have the help of a caregiver to fill in the questionnaire. When excluding this group of patients, since the reported data maybe less reliable, both medication problems during admission (p = 0.00, odds-ratio 6.0, 95%-confidence interval 2.4-14.9) and a LED-value of more than 600 mg/dag (p = 0.024, odds-ratio 3.25, 95%-confidence interval 1.2-9.0) are significant risk factors for deterioration, and infections aren't (p = 0.08).

When adjusting for possible confounders (logistic regression was applied using the following variables: Age, gender, PD duration, LED-value, Hoehn& Yahr scale, presence of cognitive problems, recruitment centre, wrong medication distribution, complications, infections, surgery, non-neurologic ward admission, consultation of PD nurse specialist and involvement of paramedics), there was still a significantly increased risk of deterioration in PD patients who had received incorrect medication (P = 0.042).

### Validation

We were able to retrieve clinical files of 84 (52%) admissions. Most of the other files got lost because of an intermittent change in computerized medical systems. In those files, which thus comprise a sample half the size of our patient sample, a doctor only once documented deterioration of PD. There was no report of deterioration by a nurse (vs. 34 by the patients). PD medication distribution problems were mentioned 7 times by a doctor and 12 times by a nurse (vs. 42 by the patients). Urinary tract infections were reported 8 times (vs. 12), confusion 13 times (vs. 35), pneumonia 3 times (vs. 4) and furthermore 3 others.

## Discussion

We sought to assess the prevalence and risk factors of deterioration in hospitalized PD patients, as evidence suggests that a substantial proportion of PD patients actually worsen when admitted to a hospital [1,2]. In our population of 684 PD patients almost one fifth had been hospitalized in the last year. Traumatic injury, infections, direct PD-related problems, and problems with the circulatory and digestive system were the main admission reasons, which accords with prior literature [1,2]. As in those studies, confusion and infections were the most common complications during hospitalization [1].

To our knowledge this is the first study systematically analysing different risk factors for deterioration of PD patients both for admissions with and without surgery.

There have been earlier studies documenting high rates of incorrect medications given to hospitalized PD patients, some as high as 74%. All these, on surgical wards and on Accident & Emergency departments, found that this was associated with deterioration, but to varying degrees. All these studies were retrospective, and selection of the patient sample was unclear [3-5]. We found having surgery or not did no matter in terms of medication distribution problems or complications. Somewhat unexpected, neurology wards do not do better, as there was no statistically significant difference between different wards regarding problems with medication distribution, complications, and PD deterioration.

There is one retrospective study suggesting that pre-operative or immediate post-operative neurological consultation of PD patients having surgery may result in higher post-operative improvement of total Unified Parkinson's Disease Rating Scale with most effect on activities on daily living [6]. In our study PD nurse specialists (as part of the movement disorder teams) were involved in a quarter of the admissions on a non-neurological ward. This was associated with a higher risk on deterioration during these admissions. This is probably reverse causation, since PD nurse specialists were asked to see the patient when deterioration had already occurred.

Second to medication distribution problems with a 5.8 higher risk on deterioration, complications are significantly related to PD deterioration, with infections as mean factor with an increased risk of 6.7. Paramedic care did not appear to be of influence. When analysing different patient and PD related factors in relation to deterioration, only a LED-value above > 700 mg/day showed to be a significant risk factor. For higher age and higher Hoehn and Yahr scores there was a tendency towards, but not a significantly, higher risk. When excluding those patients who had no help with answering the questionnaire and had cognitive problems, only wrong medication distribution and a LED-value of more than 600 mg/dag are significant risk factors.

There are significant differences for some variables between the hospitals which can be expected since the Maastricht University Medical Centre is, unlike the others, an university hospital (with more complex PD patients and more patients with deep brain stimulation). There is however no significant difference between the centres in medication distribution problems.

When correcting for different variables, including those that were significant different between the three centres, wrong medication distribution is the most important significantly increased risk factor for deterioration. Comparing our data with data on medication errors in hospitalized patients in general, showing medication errors on average in 6 per 100 hospitalized patients, this study supports the higher vulnerability of PD patients [10].

When validating the reported data by PD patients with clinical files of the admissions there seems to be mainly a strong underreporting of deterioration of PD supporting the lack of knowledge of this problem.

Apparently much more needs to be done to prevent incorrect medication distribution and complications. Better education of health care professionals, both on a neurological and non-neurological wards, to stress the importance of correctly administrated PD drugs and to prevent complications might result in less deterioration. Rigid electronic medication systems in hospitals do not seems to support home schedules of PD medication. Self-administration of PD drugs by able patients could be an option. The effects of an electronic warning system to alert the treating team of the vulnerability of this patient group, and a multidisciplinary approach, with a role for the clinical pharmacist and movement disorder team, should be evaluated in future studies.

This study has a number of limitations. Information was asked about the previous year, causing possible recall bias. Medication administration was assessed through self-report, and patients who died during admission were obviously not included. Since it was not possible to uncover adverse medication prescription during the admissions this aspect was not taken into account. Further studies should be undertaken to shed more light on these aspects. Nevertheless, we believe that these limitations do not invalidate our conclusions.

## Conclusions

This is the first study systematically analysing different risk factors for deterioration of hospitalized PD patients both for admissions with and without surgery. There is a high rate of deterioration during hospitalization of PD patients on all wards. Especially incorrect medication distribution, but also infections are related to this. Measures should be taken to improve care and should be incorporated in guidelines.

## Competing interests

Oliver H.H. Gerlach, Martijn P.G. Broen and Ad J. Vermeij do not have any financial or other conflict of interest. Peter H.M.F. van Domburg has served on scientific advisory boards for Novartis and Lundbeck Inc. Wim E.J. Weber is European research editor of British Medical Journal.

## Authors' contributions

OHHG and WEJW participated in design, data collection, interpretation of the data, and prepared the manuscript. MPGB participated in data collection, interpretation of the data, and prepared the manuscript. PHMFD and AJV participated in data collection and helped to bring the manuscript to its final version. All authors read and approved the final manuscript.

## Pre-publication history

The pre-publication history for this paper can be accessed here:

http://www.biomedcentral.com/1471-2377/12/13/prepub

## Supplementary Material

Additional file 1 **Introduction letter for questionnaire**. Introduction letter for PD patients for the questionnaire. Click here for file

Additional file 2 **Questionnaire**. Questionnaire for PD patients. Click here for file
