# Supplementary material for: Deterioration of Parkinson's disease during hospitalization: survey of 684 patients
Source: BMC Neurol. 2012 Mar 8;12:13. doi: 10.1186/1471-2377-12-13 (PMC3314577; doi:10.1186/1471-2377-12-13)
Supplement: Additional file 1 — Introduction letter for questionnaire. Introduction letter for PD patients for the questionnaire. [file 1471-2377-12-13-S1.DOC]

Dear Sir/Madam,

You are being treated for Parkinson’s disease in our hospital and/or you use drugs that are prescribed for this disease.

Many patients with Parkinson’s disease have told us that they have deteriorated after a hospital stay. With this survey we would like to know your experience in this. With this information we strive to improve the quality of our patient care.

Therefore we would very much appreciate your cooperation to complete the attached questionnaire.

*Confidential data processing*

Personal information is strictly confidential and processed completely anonymously. Nobody will be able to determine on the basis of your answers who gave these answers.

## Voluntary participation

Participation to this research is completely voluntary. You may refuse to participate without given a reason. Of course this will not influence your further treatment.

## Questionnaire

You will notice that the questions resemble the questions normally asked by your treating doctor. We would *prefer* that you answer the questionnaire yourself (the patient), together with, if applicable, your partner or caregiver.

When answering the questions in case you are not the patient, all questions do relate to the patient.

Could you please return the questionnaire within two weeks. There is an envelope attached, there is no stamp required. You can also hand it over at the outpatient clinic.

If you have any questions or remarks concerning this survey please contact us: telephone number.

We try to maintain our address file as accurately as possible. Obviously, this questionnaire might have been delivered to you by mistake. It is also possible that this survey is sent to you on a very inappropriate moment for you or your family. If so, we apologize.

Thank you for your cooperation.
