# Supplementary material for: Deterioration of Parkinson's disease during hospitalization: survey of 684 patients
Source: BMC Neurol. 2012 Mar 8;12:13. doi: 10.1186/1471-2377-12-13 (PMC3314577; doi:10.1186/1471-2377-12-13)
Supplement: Additional file 2 — Questionnaire. Questionnaire for PD patients. [file 1471-2377-12-13-S2.DOC]

A. GENERAL INFORMATION

1. Who answers this questionnaire?

0 Patient

0 Patient with the help of partner or caregiver

0 Partner and/or caregiver

0 Other, namely ………………….(please mention the relationship with the patient)

2. What is your date of birth? (of the patient):

……...-……….-……… (day-month-year)

3. What is your gender?

0 Male

0 Female

4. What is your civil status?

0 Living on your own

0 Living together with partner, not married

0 Living together with somebody else than partner, namely …………………..

0 Married

0 Widower/Widow

0 Divorced

0 Other, namely ………………………….................

5. What is your nationality?

0 Dutch

0 Belgium

0 German

0 Turkish

0 Moroccan

0 Other, namely ………………………….................

#### B. CARE RELATED QUESTIONS

6. Have you been admitted to a hospital in the previous year?

0 No (go to question 18)

0 Yes, namely:

- Please mention all admissions both for neurology and other specialties, also if there were multiple admissions per specialty.

- Please mention for every admission if you did have surgery or not (cross out what is not applicable at ‘Yes / No’)

- Please mention all complications, if any, even if there were more than one per admission. Please mention if this complication was before and/or after a possible surgery (cross out what is not applicable at ‘Before / after/ before and after’).

(*Examples of complications:* Confusion, hallucinations, pneumonia, urinary tract infection, wound infections, thrombosis, falls, worsening of motor function, mood disorders, pulmonary embolism, memory impairment etc.)

*Example 1* ..*Internal medicine….* ……*pneumonia……*.….. Yes / No …no complication… Before/after/before and after

*2* ..*Surgery…*…………. ……*hipfracture*……….. Yes / No …thrombosis….…... Before/after/before and after

....confusion……..… Before/after/before and after

**Admission number** Specialty illness/complaint Surgery Complication(s) Before and/or after a possible surgery

**1** …………………….. ……..…………………… Yes / No …………………… Before/after/before and after

…………………… Before/after/before and after

…………………… Before/after/before and after

**2** …………………….. ……..…………………… Yes / No …………………… Before/after/before and after

…………………… Before/after/before and after

…………………… Before/after/before and after

**3** …………………….. ……..…………………… Yes / No …………………… Before/after/before and after

…………………… Before/after/before and after

…………………… Before/after/before and after

**4** …………………….. ……..…………………… Yes / No …………………… Before/after/before and after

…………………… Before/after/before and after

…………………… Before/after/before and after

**5** …………………….. ……..…………………… Yes / No …………………… Before/after/before and after

…………………… Before/after/before and after

***The following questions can refer to an ‘admission number’, you can find it at question 6.***

7. Was Parkinson’s disease medication distributed according to the same schedule as you take your medication at home? (more than one answer possible)

0 Not applicable, no admission

0 Yes

0 No, the time of medication distribution was not the same at admission number(s) …………... (see question 6)

0 No, the kind of Parkinson’s disease medication did not match at admission number(s)………….(see question 6)

0 Don’t know

0 Other answer, namely ………………………….................

8. Was there during one of the admissions an interruption of Parkinson’s disease medication? (more than one answer possible)

0 Not applicable, no admission

0 No

0 Yes, during a surgery, namely at admission number(s) ……….....(see question 6)

0 Yes, this was on purpose namely because of …………………………………at admission number(s)……..(see question 6)

0 Yes, this was not on purpose, namely at admission number(s) …………….(see question 6)

0 Don’t know

0 Other answer, namely ………………………….................

9. Did you have to point out to the personnel that there was a problem with the distribution of Parkinson’s disease medication? (more than one answer possible)

0 Not applicable, no admission

0 No

0 Yes, point out incorrect distribution time, namely at admission number(s)……………(see question 6)

0 Yes, point out incorrect Parkinson’s disease medication, namely at admission number(s) ……...(see question 6)

0 Yes, point out that Parkinson’s disease medication was forgotten, namely at admission number(s) ……...(see question 6)

0 Don’t know

0 Other answer, namely ………………………….................

10. Did you have to point out to the personnel that there was a problem with the distribution of Parkinson’s disease medication after a possible surgery? (more than one answer possible)

0 Not applicable, no surgery

0 No, I did have surgery but there was no problem with the medication distribution after the surgery at admission number(s) ……...(see question 6)

0 Yes, point out incorrect Parkinson’s disease medication, namely at admission number(s) ……...(see question 6)

0 Yes, point out that Parkinson’s disease medication was forgotten, namely at admission number(s) ……...(see question 6)

0 Yes, point out that there was incorrect Parkinson’s disease medication , namely at admission number(s) ……...(see question 6)

0 Don’t know

0 Other answer, namely ………………………….................

11. Was there a deterioration of Parkinson’s disease (i.e. decline in motor function) during the admission? (more than one answer possible)

0 Not applicable, no admission

0 No, no deterioration (go to question 14)

0 Yes, namely ……………………………………………(please point out what aspect deteriorated) at admission number(s) ……...(see question 6)

0 Don’t know

0 Other answer, namely ………………………….................

12. Did this deterioration start after a possible surgery? (more than one answer possible)

0 Not applicable, no surgery

0 Not applicable, no deterioration

0 No, deterioration started already before surgery, namely at admission number(s) ……...(see question 6)

0 Yes, deterioration started after surgery, namely at admission number(s) ……...(see question 6)

0 Don’t know

0 Other answer, namely ………………………….................

13. Did this deterioration start after interruption of Parkinson’s disease medication?

0 Not applicable, no admission

0 Not applicable, no deterioration

0 No

0 Yes, namely at admission number(s) ……...(see question 6)

0 Don’t know

0 Other answer, namely ………………………….................

14. Was there a deterioration of Parkinson’s disease after the admission?

0 Not applicable, no admission

0 No, no deterioration

0 Yes, namely ……………………………………………(please point out what aspect deteriorated) at admission number(s) ……...(see question 6)

0 Don’t know

0 Other answer, namely ………………………….................

15. How long did the deterioration of Parkinson’s disease last? (more than one answer possible)

0 Not applicable, no admission

0 No deterioration during or after the admission at admission number(s) ……...(see question 6)

0 Deterioration lasted for: (if deterioration during or after more than one admission please mention the duration of all of them)

…….days, namely at admission number(s) ……...(see question 6)

…….weeks

…….months

…….days, namely at admission number(s) ……...(see question 6)

…….weeks

…….months

…….days, namely at admission number(s) ……...(see question 6)

…….weeks

…….months

0 No full recovery from deterioration, namely at admission number(s) ……...(see question 6)

0 Don’t know

16. Was there contact with a Parkinson’s disease nurse specialist or another allied healthcare facility during the admission? (for example occupational therapist, speech therapist, etc.)

0 Not applicable, no admission

0 No

0 Don’t know

0 Yes, namely :

0 Parkinson’s disease nurse specialist at admission number(s) ……...

(see question 6)

0 Physical therapy at admission number(s) ……...(see question 6)

0 Occupational therapy at admission number(s) ……...(see question 6)

0 Speech therapy at admission number(s) ……...(see question 6)

0 Psychology at admission number(s) ……...(see question 6)

0 Social worker at admission number(s) ……...(see question 6)

0 Another allied healthcare facility, namely:

*Allied healthcare facility* admission number(s): see question 6

…………………. ………

…………………. ………

17. Do you use more frequently allied healthcare facilities after the admission compared to before?

0 Not applicable, no admission

0 Don’t know

0 No

0 Yes, namely :

0 Parkinson’s disease nurse specialist at admission number(s) ……...

(see question 6)

0 Physical therapy at admission number(s) ……...(see question 6)

0 Occupational therapy at admission number(s) ……...(see question 6)

0 Speech therapy at admission number(s) ……...(see question 6)

0 Psychology at admission number(s) ……...(see question 6)

0 Social worker at admission number(s) ……...(see question 6)

0 Another allied healthcare facility, namely:

*Allied healthcare facility* admission number(s): see question 6

…………………. ………

…………………. ………

18. Where do you live now?:

0 Independent house

0 Assisted living

0 Elderly home

0 Nursing home

0 Other answer, namely ………………………….................

19. If you use medication, do you take the medication as prescribed by the doctor? (more than one answer possible)

0 Yes, the correct medication at the correct times

0 No, I don’t take the medication at all

0 No, I sometimes forget medication

0 No, I often forget medication

0 No, I don’t take the medication at the prescribed times because ……………………….

0 No, I often take extra medication because of …………………………………………..

0 No, I often don’t take medication because of side effects

0 No, I often don’t take medication because of the costs

0 No, I often don’t take medication because I have to many tablets

0 Other answer, namely ………………………….................

#### C. DISEASE RELATED QUESTIONS

20. Do you have (had) tremors? (more than one answer possible)

0 No

0 Yes, the head

0 Yes, the arms/hands

0 Yes, the legs/feet

0 Other answer, namely ………………………….................

21. Do you have (had) stiffness/rigidity? (more than one answer possible)

0 No

0 Yes, the arms

0 Yes, the legs

0 Other answer, namely ………………………….................

22. Do you have (had) bradykinesia or did you become slower because of Parkinson’s disease?

0 No

0 Yes

23. Do you have (had) postural instability?

0 No

0 Yes, but I never fall

0 Yes, I fall sometimes

0 Yes, I fall often

0 Yes, I can not walk anymore

0 Other answer, namely ………………………….................

24. If you fall, how often do you fall on average per month?

…….. times on average per month

25. In what year did the symptoms (as described in question 20 to 24) of Parkinson’s disease begin?

0 In the year : ……………..

0 Not applicable

0 Don’t know

26. Do you currently have one or more of the following symptoms? (more than one answer possible)

0 Problems with turning over in bed

0 Reduced smell

0 Problems with writing

0 Problems with using cutlery

0 Joint pain

0 Back pain

0 Cramps

0 Pain, namely …………………

0 Changed sexual behaviour, namely …………..

0 Psychosis/hallucinations

0 Dyskinesia

0 On-off moments

0 Depressed mood

0 Anxiety and/or panic attacks

0 Light-headedness when standing quickly

0 Swallow problems

0 Choking

0 Problems with urination

0 Problems with defecation

0 Memory problems

0 Behaviour problems

0 Sensation of tingling (paresthesia)

0 Sleep disorders

0 Problems with breathing, namely …………..

0 Other answer, namely ………………………….................

27. Which of the following answers fits you the most? (just one answer possible)

0 No signs of the disease

0 Unilateral symptoms only, no impairment of balance

0 Bilateral symptoms, no impairment of balance

0 Mild to moderate disease symptoms, balance impairment, physically independent

0 Severe disability, but still able to walk or stand unassisted

0 Needing a wheelchair or bedridden unless assisted

28. Do you currently take medication?

0 Yes

0 No (go to question 30)

29. What medication do you currently take? (all drugs for all diseases)

Please also mention any side effects.

Optionally, a copy of your medication prescriptions can be added.

*(Example: sinemet 125mg 3 times per day 2 tablets Drowsiness)*

Name of drugs: Dose How many times a day Side effect

how many tablets

1 ………………… ……… ………………………… ……………………………….

2 ………………… ……… ………………………… ……………………………….

3 ………………… ……… ………………………… ……………………………….

4 ………………… ……… ………………………… ……………………………….

5 ………………… ……… ………………………… ……………………………….

6 ………………… ……… ………………………… ……………………………….

7 ………………… ……… ………………………… ……………………………….

8 ………………… ……… ………………………… ……………………………….

9 ………………… ……… ………………………… ……………………………….

10...……………… ……… ………………………… ……………………………….

11…………...…… ……… ………………………… ……………………………….

12 ..……………… ……… ………………………… ……………………………….

13 ..……………… ……… ………………………… ……………………………….

14 ..…………...… ……… ………………………… ……………………………….

15...……………… ……… ………………………… ……………………………….

30. Do you have any questions or remarks concerning this survey?

……………………………………………………………………………………………………………………………………………………………………………………………………

…………………………………………………………………………………………………

**END OF QUESTIONNAIRE**

**Could you please verify that you have answered all the questions completely?**
